# Association of Beneficiary-Level Risk Factors and Hospital-Level Characteristics With Medicare Part B Drug Spending Differences Between 340B and Non-340B Hospitals

**DOI:** 10.1001/jamanetworkopen.2022.0045

**Published:** 2022-02-18

**Authors:** Yufei Li, Susan Xu

**Affiliations:** 1 Association of American Medical Colleges, Washington, DC; 2 Partnered Evidence-based Policy Resource Center, Veterans Affairs Boston Healthcare System, Boston, Massachusetts; 3 Department of Health Law, Policy and Management, Boston University School of Public Health, Boston, Massachusetts; 4 McDermott + Consulting, Washington, DC

## Abstract

**Importance:**

Critics of the federal 340B Drug Pricing Program raised concerns that the program might provide financial incentives for participating hospitals to prescribe more and/or more expensive drugs because the revenue generated from Medicare reimbursement exceeds the purchase price by a substantial margin. Studies showing higher Medicare Part B drug spending at hospitals that are 340B hospitals, which can purchase outpatient drugs from manufacturers at discounted prices, compared with non-340B hospitals were used by the Centers for Medicare & Medicaid Services to justify their 340B payment policy that reduced Medicare payments for drugs in the 340B program in 2018 and thereafter. The Centers for Medicare & Medicaid Services attributed higher spending to the 340B benefit and believed that payment cuts would reduce the financial incentives associated with higher spending. However, the lack of sufficient risk adjustments is a significant concern of study validity.

**Objective:**

To examine whether per-beneficiary Medicare Part B drug spending is significantly different between 340B and non-340B hospitals while adequately controlling for patient-level and hospital-level risk factors.

**Design, Setting, and Participants:**

A cross-sectional study was conducted from October 1, 2020, to May 30, 2021, using 2017 administrative claims data from a random 5% sample of Medicare fee-for-service beneficiaries. Included beneficiaries had at least 1 separately payable non–pass-through drug claim in 2017, were fully enrolled in Part A and Part B through 2017, and did not die in 2017.

**Main Outcomes and Measures:**

The outcome was separately payable Part B drug spending.

**Results:**

The sample included 35 364 beneficiaries (21 825 women [61.7%]; 29 996 White patients [84.8%]; mean [SD] age, 70.6 [12.0] years) and 2446 hospitals. A total of 918 hospitals (37.5%) were in the 340B program and 938 hospitals (38.3%) were teaching hospitals. There was a higher percentage of teaching hospitals among 340B hospitals (517 of 918 [56.3%]) than non-340B hospitals (421 of 1528 [27.6%]), and beneficiaries who went to 340B hospitals were more likely to be non-White than those who went to non-340B hospitals (3360 of 19 139 [17.6%] vs 1583 of 13 710 [11.5%]). The Part B drug spending difference between 340B and non-340B hospitals was not statistically significant after controlling for beneficiary-level risk factors and hospital-level characteristics ($568; 95% CI, −$283 to $1419; *P* = .19).

**Conclusions and Relevance:**

The results show that the differences in patient population and hospital-level characteristics may explain drug spending differences between 340B and non-340B hospitals, which raises doubt about the financial incentive theory of the 340B program drug discount and the justification for the Centers for Medicare & Medicaid Services’s 340B payment policy.

## Key Points

**Question:**

Is per-beneficiary Medicare Part B drug spending significantly different at hospitals that are part of the federal 340B Drug Pricing Program, which can purchase outpatient drugs from manufacturers at discounted prices, compared with hospitals that are not part of the 340B program, after adequate risk adjustments?

**Findings:**

In this cross-sectional study that included 35 364 beneficiaries and 2446 hospitals, there was no statistically significant difference in Medicare Part B drug spending between 340B hospitals and non-340B hospitals, after controlling for beneficiary-level risk factors and hospital-level characteristics.

**Meaning:**

These findings raise doubt about the financial incentive theory of 340B program drug discounts and the Centers for Medicare & Medicaid Services’ 340B drug payment policy rationale.

## Teaser

This cross-sectional study examines whether a difference exists in Medicare Part B drug spending between hospitals that are part of the federal 340B drug pricing program and those that are not part of the 340B program, while adequately controlling for patient-level and hospital-level risk factors.

## Introduction

Created in 1992 by Congress, the 340B Drug Pricing Program allows qualifying providers, generally hospitals, specialty clinics, and their associated outpatient facilities serving uninsured and low-income patients in rural communities, to obtain discounted prices on outpatient drugs from drug manufacturers.^1^ The program intends to provide financial support to covered entities so that they could use resources generated from the drug discounts to expand or improve care for low-income and vulnerable patients.^2^

Critics of the program, including the pharmaceutical industry, raised concerns that 340B program drug discounts might have unintended negative consequences that contradict the program’s original intent.^1^ Data showing higher per-patient outpatient drug spending at 340B hospitals than at non-340B hospitals^3^ has drug manufacturers and payers (eg, Medicare) questioning whether the program incentivizes participating hospitals to prescribe more and/or more expensive medicines to increase the margin between revenue generated from the reimbursement by insurers and the discounted 340B program drug prices paid for outpatient drugs. Analysis by the Medicare Payment Advisory Commission (MedPAC) showing substantial growth of the 340B program has fueled further concern among policy makers that the program might have been associated with the growth of Medicare Part B drug spending,^1^ although the growth rate also likely is associated with multiple factors, including increasing drug prices,^4^ patient factors, the expansion of 340B program eligibility criteria,^1,4,5^ and the shift of care from inpatient to outpatient settings.^6^

Starting in 2018, the Centers for Medicare & Medicaid Services (CMS) reduced the Medicare payments for separately payable outpatient drugs acquired by 340B facilities by 28.5%, often referred to as *CMS 340B payment policy*.^4^ Medicare Part B covers a specific set of drugs normally administered by infusion or injection in hospital outpatient departments or physician offices, which constituted 20.3% of Medicare drug spending in 2019.^7,8^ This payment reduction on 340B hospitals was estimated to be approximately $1.6 billion in 2018 and then was redistributed in a budget-neutral manner to all hospitals paid under the Outpatient Prospective Payment System.^4^ The CMS 340B payment policy reflected concerns that the 340B drug discounts might be associated with an increase in Part B drug spending and sought to remove or reduce any associated financial incentives. The CMS cited 2 reports in support of their policy. One was the US Government Accountability Office (GAO) June 2015 report indicating higher per-beneficiary Medicare Part B drug spending at 340B Disproportionate Share Hospitals (DSHs) than at non-340B hospitals ($58 vs $27 in 2008; $144 vs $60 in 2012).^3^ The CMS also cited MedPAC’s May 2015 report to Congress that showed that, from 2008 to 2012, Medicare Part B drug spending for chemotherapy drugs and drug administration increased faster among 340B DSHs than among non-340B hospitals (19.1% vs 13.9%).^1^

However, these cited reports are flawed because they do not adequately adjust for confounding factors, such as beneficiary-level risk factors and hospital-level characteristics. In MedPAC’s analysis, neither patient risk, such as age, chronic conditions, and comorbidities, nor hospital-level characteristics, such as teaching status, ownership, and geographical area, were evaluated.^1^ Although the GAO’s analysis included several hospital-level characteristics and hospital-level mean patient risk scores, no risk factors, including patient clinical conditions, were examined at the patient level.^3^

Failing to sufficiently account for confounding factors may lead to a false association. For example, before making any risk adjustments, Kalidindi et al^9^ found higher per-beneficiary chemotherapy drug spending at hospital outpatient departments than physician offices. However, controlling for highly relevant factors, such as cancer type and previous health status, produced the opposite results. Similarly, after examining drug spending by clinical condition, MedPAC found that mean drug spending varied by drug type.^10^ Specifically, they reported that per-beneficiary spending levels at 340B hospitals were similar to those at non-340B hospitals for 3 of the 5 types of cancer examined, and higher spending levels at 340B hospitals appeared to be specific to lung cancer and prostate cancer, for which 340B hospitals tended to use more new therapies (eg, immunotherapy) with higher per-unit costs.^10^ MedPAC found that associations of the 340B program with cancer drug spending were likely to be idiosyncratic and not generalizable to other cancer types or conditions.^10^

The primary aim of our study is to evaluate the association of beneficiary-level risk factors and hospital-level characteristics with the difference in Medicare Part B drug spending between 340B and non-340B hospitals. Specifically, we hypothesize that higher mean spending per beneficiary of separately payable Medicare Part B drugs at 340B hospitals reflects both the more complex patient populations treated at 340B hospitals and the hospital-level characteristics that tend to attract patients with unmeasured risks. We also hypothesize that, after controlling for beneficiary-level risk factors and hospital-level characteristics, the observed higher spending level will no longer be statistically significant. Our study will help inform the evaluation of the 340B program and the CMS 340B payment policy.

## Methods

### Data Sources and Study Population

For this study, conducted from October 1, 2020, to May 30, 2021, we used 2017 administrative claims data from a random 5% sample of Medicare beneficiaries. We chose data from 2017 because it was the year prior to the implementation of CMS 340B payment policy, which altered the financial benefits of the 340B program that might cause behavior change among 340B hospitals. We used 2017 Medicare fee-for-service Part B claims to identify separately payable Part B drugs and Medicare payment amounts for drugs. We used the 2017 Medicare Master Beneficiary Summary File to capture beneficiary-level demographic characteristics (age, sex, and race and ethnicity) and enrollment status. We obtained the race and ethnicity of patients from the CMS Medicare Master Beneficiary Summary File, which is a deidentified data set. The category of race and ethnicity is defined in the data set, which includes Asian, Black, Hispanic, North American Native, other, and unknown. In our analyses, we recategorized race and ethnicity as a binary variable (White vs non-White). Non-white includes Black, other, Asian, Hispanic, North American Native, and unknown. We obtained the 2017 median household income by county data from the US Census Bureau. We extracted hospital-level characteristics, such as teaching status, urban or rural classification, and ownership from the Calendar Year 2017 Hospital Outpatient Prospective Payment System Final Rule Payment Impact File and Fiscal Year 2017 Inpatient Prospective Payment System Final Rule Impact File. We used the CMS Hierarchical Condition Categories model to identify beneficiary clinical conditions and included all claims for services rendered to the beneficiary in 2017 when running the model. We obtained hospitals’ 340B status from the Health Resources and Services Administration website. Several types of entities are covered in the 340B program, including Health Resources and Services Administration–supported health centers and lookalikes (community-based health care providers that meet the requirements of the Health Resources and Services Administration Health Center Program, but do not receive Health Center Program funding), Ryan White clinics and state AIDS drug assistance programs, Medicare and Medicaid DSHs, children’s hospitals, and other safety net providers.^11^ We focused our study only on 340B hospitals eligible through the 340B DSH program, which accounts for most of the discounted drug purchases made through the 340B Drug Pricing Program,^3^ and we compared them with non-340B hospitals. This study followed the Strengthening the Reporting of Observational Studies in Epidemiology (STROBE) reporting guideline and was exempt from institutional review board review by the Association of American Medical Colleges because it was not human participants research, as defined in 45 CFR §46. This study was considered a secondary analysis of deidentified data with no interaction with human participants.

We included only beneficiaries who had at least 1 separately payable non–pass-through drug claim in 2017. We further restricted the sample to beneficiaries who were fully enrolled in Part A and Part B through 2017 and excluded those who died in 2017, to ensure that all the beneficiaries had 12 months of coverage. In addition, we excluded beneficiaries enrolled in Medicare Advantage plans because their drug spending data were not available.

### Statistical Analysis

We conducted 2 phases of ordinary least-squares regression models. The first phase was a beneficiary-level regression, and the second phase used the output from the first phase in hospital-level regression analyses. The models describe how we used the estimated annual Part B drug spending from the beneficiary-level regression to compute the dependent variable of the hospital-level regression.

In phase 1, we fitted a beneficiary-level multivariate ordinary least-squares regression to estimate annual separately payable Part B drug spending (hereafter referred to as *drug spending*) for each beneficiary after controlling for beneficiary-level characteristics. The model is as follows: Drug_Spending*_i_*_,_*_t_* = α + βBene_Char*_i_*_,_*_t_* + ε*_i_*_,_*_t_*, where Drug_Spending*_i_*_,_*_t_* is the observed drug spending for beneficiary *i* in year *t* (*t* = 2017) aggregated from drug spending at each drug administration and Bene_Char is a vector including the following beneficiary-level characteristics: age, sex, race and ethnicity, the median income of the county where the beneficiary resided in 2017, current reason for Medicare enrollment (age, disability, end-stage renal disease, and both disability and end-stage renal disease), dual eligibility for Medicare and Medicaid, and 87 beneficiary clinical conditions based on the Hierarchical Condition Categories model (eg, diabetes with chronic complications, congestive heart failure, and vascular disease). Phase 1 of the model intentionally excluded hospital-level characteristics so that the residual in the regression included spending potentially associated with hospitals, to use in phase 2 regressions.

In phase 2, we examined associations between hospital-level characteristics and hospital spending variation. To implement this analysis, we first computed beneficiary-level excess spending. Using the regression coefficients (β) and beneficiary-level characteristics (Bene_Char) in the phase 1 regression model, we computed estimated beneficiary-level drug spending (hereafter referred to as beneficiary-level Drug_Spending*_i,t_*), which is the expected spending based on beneficiary-level characteristics. The difference between Drug_Spending*_i_*_,_*_t_* and beneficiary-level Drug_Spending*_i_*_,_*_t_* is henceforth referred to as *excess spending* and represents the part of beneficiary drug spending not explained by beneficiary-level characteristics and potentially explained by hospital-level characteristics. The measure can take positive values (meaning higher-than-expected spending) or negative values.

Next, we allocated the beneficiary-level excess spending to hospitals and constructed a hospital-level excess spending variable. First, we calculated hospital-level observed drug spending as the sum of all Part B drug spending at the hospital in 2017 divided by the unique number of beneficiaries who received Part B drugs in the hospitals. We conducted a *t* test comparing hospital-level observed drug spending between 340B and non-340B hospitals to assess their difference in drug spending without controlling for any risk factors.

Hospital-level estimated drug spending was constructed using beneficiary-level estimated drug spending (ie, beneficiary-level Drug_Spending*_i_*_,_*_t_*) from the phase 1 regression. Because some beneficiaries received Part B drugs from multiple hospitals during the year, we first computed a hospital’s share of a beneficiary’s spending based on the portion of a beneficiary’s observed spending contributed by the hospital. Beneficiary-level Drug_Spending*_i_*_,_*_t_* was allocated to each contributing hospital using the hospital’s spending share. Similar to how we computed hospital-level observed drug spending, hospital-level estimated drug spending is the sum of all beneficiary-level estimated drug spending allocated to the hospital divided by the unique number of beneficiaries who received Part B drugs in the hospitals in 2017.

Phase 2 consisted of 2 hospital-level ordinary least-squares regressions, 1 single-variable regression, and 1 multivariate regression, used to evaluate the associations of a hospital’s 340B status and hospital-level excess drug spending before and after adjusting for other hospital-level characteristics. We fitted the 2 regressions as Excess_Spending*_j_*_,_*_t_* = α + β340B_Status*_j_*_,_*_t_* (model 1) and Excess_Spending*_j_*_,_*_t_* = α + β340B_Status*_j_*_,_*_t_* + γOther_Hosp_Char*_j_*_,_*_t_* (model 2), where Excess_Spending*_j_*_,_*_t_* was the difference between observed and estimated hospital-level drug spending for hospital *j* at year *t* (*t* = 2017). A positive value denotes that the hospital had higher observed drug spending per beneficiary than their beneficiary risk-adjusted estimated spending level. We selected hospital-level characteristics for hospital-level multiple linear regression based on previous studies of hospital spending, including teaching status (measured by intern and resident to bed ratio),^12^ urban or rural classification (large urban, other urban, and other),^13^ number of beds,^14^ and ownership (voluntary, proprietary, and government).^15^ The simple linear regression was used to assess the association of 340B status with hospital-level drug spending after controlling for beneficiary-level risk factors, and the multiple linear regression was used to examine whether hospital 340B status was still associated with higher drug spending after further controlling for other hospital-level characteristics. The disproportionate patient percentage (DPP) is often used in the analysis of hospital spending; however, because the DPP is used to compute DSH payment adjustment and thus establish 340B eligibility criteria, we ran a separate linear regression to examine its association with hospital-level spending.

We used SAS software, version 9.4 (SAS Institute Inc) to conduct our analysis. All statistical tests were 2-sided and considered statistically significant at *P* < .05.

## Results

The final sample consisted of 35 364 beneficiaries (21 825 women [61.7%]; 29 996 White patients [84.8%]; mean [SD] age, 70.6 [12.0] years) and 2446 hospitals based on the observation of 141 392 claims with Part B drug payment. Beneficiaries who went to 340B hospitals were younger (mean age, 70.0 years [95% CI, 69.8-70.1 years] vs 71.7 years [95% CI, 71.5-71.9 years]), more likely to be non-White (3360 of 19 139 [17.6%] vs 1583 of 13 710 [11.5%]) and dually eligible for Medicare and Medicaid (3889 of 19 139 [20.3%] vs 2186 of 13 710 [15.9%]), and less likely to enroll because of old age (15 382 of 19 139 [80.4%] vs 11 644 of 13 710 [84.9%]) (Table 1) and had more clinical conditions (eTable 1 in the Supplement) than beneficiaries who went to non-340B hospitals. A total of 918 hospitals (37.5%) were in the 340B program. There were higher percentages of teaching hospitals (517 of 918 [56.3%] vs 421 of 1528 [27.6%]), urban hospitals (777 of 918 [84.6%] vs 1079 of 1528 [70.6%]), and voluntary hospitals (709 of 918 [77.2%] vs 913 of 1528 [59.8%]) among 340B hospitals than non-340B hospitals. On average, 340B hospitals had a higher intern and resident to bed ratio (0.14 [95% CI, 0.12-0.15] vs 0.04 [95% CI, 0.03-0.04]), a higher DPP (0.39 [95% CI, 0.38-0.39] vs 0.25 [95% CI, 0.24-0.25]), and more beds (303 [95% CI, 288-319] vs 172 [95% CI, 165-180]) than non-340B hospitals (Table 2).

**Table 1.** Demographic Characteristics of Beneficiaries by Hospital 340B Status, 2017

Characteristic	Beneficiaries administered Medicare Part B drugs, No. (%)			*P* value^b^
	All (N = 35 364)	At 340B hospitals only (n = 19 139)^a^	At non-340B hospitals only (n = 13 710)^a^	
Age, mean (95% CI)	70.6 (70.5-70.7)	69.9 (69.8-70.1)	71.7 (71.5-71.9)	<.001
Sex				
Male	13 539 (38.3)	7263 (37.9)	5121 (37.4)	.27
Female	21 825 (61.7)	11 876 (62.1)	8589 (62.6)	
Race and ethnicity^c^				
Non-White	5368 (15.2)	3360 (17.6)	1583 (11.5)	<.001
White	29 996 (84.8)	15 779 (82.4)	12 127 (88.5)	
County income, mean (95% CI), $	59 969 (59 798-60 140)	59 465 (59 236-59 693)	59 803 (59 528-60 078)	.06
Current reason for enrollment				
Old age	29 109 (82.3)	15 382 (80.4)	11 644 (84.9)	<.001
Both disability and ESRD	6016 (17.0)	3624 (18.9)	1981 (14.4)	
ESRD	159 (0.5)	97 (0.5)	44 (0.3)	
Disability	79 (0.2)	36 (0.2)	41 (0.3)	
Dual eligibility	5619 (15.9)	3889 (20.3)	2186 (15.9)	<.001

Abbreviation: ESRD, end-stage renal disease.

^a^ Only patients who had only gone to 1 type of hospital (n = 32 849) were included, which constitutes 92.9% of all the patients in our study.

^b^ Generated from χ^2^ tests or *t* tests.

^c^ We obtained the race and ethnicity of patients from the Centers for Medicare & Medicaid Services Medicare Master Beneficiary Summary File, which is a deidentified data set. The category of race and ethnicity is defined in the data set, which includes Asian, Black, Hispanic, North American Native, White, other, and unknown. In our analyses, we recategorized race and ethnicity as a binary variable (White vs non-White). Non-White includes Asian, Black, Hispanic, North American Native, other, and unknown.

**Table 2.** Hospital-Level Characteristics by Hospital 340B Status, 2017

Characteristic	No. (%)			*P* value^a^
	All hospitals (N = 2446)	340B hospitals (n = 918)	Non-340B hospitals (n = 1528)	
Teaching hospital	938 (38.3)	517 (56.3)	421 (27.6)	<.001
Urban or rural classification				
Large urban	970 (39.7)	383 (41.7)	587 (38.4)	<.001
Other urban	886 (36.2)	394 (42.9)	492 (32.2)	
Rural	590 (24.1)	141 (15.4)	449 (29.4)	
Ownership				
Voluntary	1622 (66.3)	709 (77.2)	913 (59.8)	<.001
Proprietary	474 (19.4)	30 (3.3)	444 (29.1)	
Government	350 (14.3)	179 (19.5)	171 (11.2)	
Intern and resident to bed ratio, mean (95% CI)	0.08 (0.07-0.08)	0.14 (0.12-0.15)	0.04 (0.03-0.04)	<.001
DPP (95% CI)^b^	0.30 (0.29-0.31)	0.39 (0.38-0.39)	0.25 (0.24-0.25)	<.001
No. of beds (95% CI)	221 (214-229)	303 (288-319)	172 (165-180)	<.001

Abbreviation: DPP, disproportionate patient percentage.

^a^ Generated from χ^2^ tests or *t* tests.

^b^ The DPP is a continuous variable used as a proxy to measure low-income and uninsured populations served by a hospital. Through a nonlinear formula, the DPP is used to compute Disproportionate Share Hospital (DSH) payment adjustment, which is also used to establish 340B eligibility criteria. Specifically, DSH hospitals must have a minimum of 11.75% DSH payment adjustment percentage to be eligible for the 340B program.

The beneficiary-level multiple linear regression (eTable 2 in the Supplement) indicated that age, sex, county income, current reason for enrollment, and dual eligibility were all significantly associated with drug spending after controlling for other beneficiary-level characteristics. The following 5 clinical conditions were associated with higher drug spending: HIV/AIDS, amyotrophic lateral sclerosis and other motor neuron diseases, morbid obesity, exudative macular degeneration, and pressure preulcer skin changes or unspecified stage. Eighteen clinical conditions were associated with lower drug spending after controlling for other beneficiary-level characteristics.

After aggregating both the observed and estimated drug spending to the hospital level, we found that the median observed drug spending was $5959 (IQR, $1383-$12 437) for all hospitals, $9326 (IQR, $3715-$14 473) for 340B hospitals, and $4139 (IQR, $957-$10 401) for non-340B hospitals. Observed drug spending was $3023 higher for 340B hospitals compared with non-340B hospitals ($10 492 vs $7469; *P* < .001). Among 340B hospitals, 41.2% (378 of 918) spent more on drugs per beneficiary than estimated compared with 29.8% (455 of 1528) among non-340B hospitals.

The dependent variable of the hospital-level regressions (ie, excess spending) was normally distributed (mean, −$1099; 95% CI, −$1465 to −$733). Results of the hospital-level simple linear regression show that, after controlling for beneficiary-level risk factors, the excess spending variable, where a positive value denotes higher-than-estimated spending, was $1516 (95% CI, $762-$2269) higher for 340B hospitals than non-340B hospitals (*P* < .001). After further controlling for hospital-level characteristics in the hospital-level multiple linear regression, the association between 340B status and higher-than-estimated spending was no longer significant ($568; 95% CI, −$283 to $1419) (Table 3). The number of beds and urban or rural classification were significantly associated with higher-than-estimated spending after controlling for other hospital-level characteristics. Specifically, after controlling for other hospital-level characteristcs, a 1-unit increase in the number of beds was associated with a $3 (95% CI, $1-$5) increase in higher-than-estimated spending, and the hospital-level characteristic of location in large urban areas was associated with $1966 lower-than-estimated spending (95% CI, −$3007 to −$926) compared with hospitals in rural areas. The DPP was not associated with higher-than-estimated spending ($1651; 95% CI, −$771 to $4074). Additional adjustment of the DPP in the hospital-level multiple linear regression did not change the results significantly (340B status, $612; 95% CI, −$325 to $1549).

**Table 3.** Association of Each Hospital-Level Characteristic and the Difference Between Observed and Estimated Hospital Drug Spending per Beneficiary After Controlling for Other Hospital-Level Characteristics, 2017 (N = 2466)^a^

Characteristic	Estimate (95% CI), $	*P* value
Hospital 340B status^b^	568 (−283 to 1419)	.19
Intern and resident to bed ratio	2083 (−317 to 4482)	.30
No. of beds	3 (1-5)	.007
Urban or rural classification		
Rural	0 [Reference]	
Large urban	−1966 (−3007 to −926)	<.001
Other urban	−369 (−1387 to 648)	.48
Ownership		
Government	0 [Reference]	
Voluntary	767 (−314 to 1848)	.16
Proprietary	−1077 (−2422 to 267)	.12

^a^ This shows the results of the hospital-level multiple linear regression analyses.

^b^ Reference group for hospital 340B status is non-340B hospital.

## Discussion

We chose 2017 data for this study to avoid the association of any potential behavior change in response to CMS 340B payment policy implemented in 2018. Before any risk adjustments, we found that 340B hospitals had higher Medicare Part B drug spending per beneficiary than non-340B hospitals, which is consistent with the findings of the GAO study. However, the spending difference we found without any risk controls was higher than that reported by the GAO because their study was based on mean drug spending among all outpatient beneficiaries no matter whether the beneficiary incurred any Part B drug spending or not.^3^ After controlling for beneficiary-level clinical conditions and other risk factors, the difference in drug spending between 340B and non-340B hospitals was reduced by half. In addition, the spending difference was no longer statistically significant after further controlling for hospital-level characteristics. Our results show that the differences in patient population and hospital-level characteristics between 340B and non-340B hospitals may explain drug spending differences. The GAO study did include several control variables, such as hospital-level mean patient risk scores based on patients’ characteristics, hospital size, teaching status, and ownership type, but all at the hospital level,^3^ which may explain the difference in findings between the GAO study and our study.

Our findings have important implications for evaluating the 340B program and CMS 340B payment policy. The drug spending difference between 340B and non-340B hospitals found in previous studies suggests that drug discounts of the 340B program incentivize 340B hospitals to prescribe more or higher-priced medicines to their patients.^4,16^ To remove the assumed financial incentives associated with the program and subsequently eliminate the drug spending difference between 340B and non-340B hospitals, the CMS adopted the 340B payment policy.^4^ However, our study shows no association between higher drug spending and 340B status after adequately adjusting for beneficiary-level and hospital-level risk factors. These results challenge both the financial incentive theory of 340B drug discounts and CMS 340B payment policy rationale.

### Limitations

Our study has several limitations. First, not all relevant, detailed patient risk factors (eg, cancer stage, patient referral status, and drug allergy history) are available in Medicare claims data. Therefore, we could not include them in our study. Second, selection bias may exist in our observational study because 340B hospitals may be inherently different from non-340B hospitals. Although we have adjusted for many hospital-level characteristics, there could still be unobserved confounders not adjusted for. Third, we could not measure financial incentives associated with 340B drug discounts explicitly. To examine the margin on drugs, besides drug payment, we also need to understand the drug acquisition cost, which was not available. It is also unlikely that data on drug pricing and acquisition costs are available to physicians at the point of care to influence their prescription decision-making. Fourth, the mechanism by which 340B drug discounts and payment policies may be associated with physician prescription behavior is worth further investigation.

## Conclusions

We found that the difference in Medicare Part B drug spending between 340B and non-340B hospitals is associated with different beneficiary-level and hospital-level characteristics. After controlling for beneficiary-level risk factors and hospital-level characteristics, the difference in Medicare Part B drug spending between 340B and non-340B hospitals was not statistically significant.

## Notes

Supplement. **eTable 1.** Beneficiary Characteristics by Hospital 340B Status, 2017 **eTable 2.** Association of Each Beneficiary Characteristic and Drug Spending After Controlling for Other Beneficiary Characteristics, 2017 (n = 35,364)
