# Errors in Methods

**DOI:** 10.1001/jamanetworkopen.2022.24469

**Published:** 2022-07-12

In the Original Investigation titled “Association of Beneficiary-Level Risk Factors and Hospital-Level Characteristics With Medicare Part B Drug Spending Differences Between 340B and Non-340B Hospitals,”^1^ published February 18, 2022, there were errors in the Methods section. In the first sentence of the second paragraph and the first sentence of the sixth paragraph of the “Statistical Analysis” subsection of the Methods section, “multivariate regression” should be changed to “multiple regression.” This article has been corrected.^1^
