# Supplementary material for: Association of Beneficiary-Level Risk Factors and Hospital-Level Characteristics With Medicare Part B Drug Spending Differences Between 340B and Non-340B Hospitals
Source: JAMA Netw Open. 2022 Feb 18;5(2):e220045. doi: 10.1001/jamanetworkopen.2022.0045 (PMC8857681; doi:10.1001/jamanetworkopen.2022.0045)
Supplement: Supplement. — eTable 1. Beneficiary Characteristics by Hospital 340B Status, 2017 eTable 2. Association of Each Beneficiary Characteristic and Drug Spending After Controlling for Other Beneficiary Characteristics, 2017 (n = 35,364) [file jamanetwopen-e220045-s001.pdf]

Supplementary Online Content

Li Y, Xu S. Association of beneficiary-level risk factors and hospital-level characteristics with Medicare Part B drug spending differences between 340B and non-340B hospitals. *JAMA Netw Open.* 2022;5(2):e220045. doi:10.1001/jamanetworkopen.2022.0045

eTable 1. Beneficiary Characteristics by Hospital 340B Status, 2017

eTable 2. Association of Each Beneficiary Characteristic and Drug Spending After Controlling for Other Beneficiary Characteristics, 2017 (n = 35,364)

This supplementary material has been provided by the authors to give readers additional information about their work.

© 2022 Li Y et al. *JAMA Network Open.*

eTable 1. Beneficiary Characteristics by Hospital 340B Status, 2017[a]

**Characteristics**	**Beneficiaries Received Part B Drug Administration at 340B hospitals only (n = 19,139)**	**Beneficiaries Received Part B Drug Administration at Non-340B hospitals only (n = 13,710)**	***P* value[b]**
Age	69.94 [69.77, 70.12]	71.73 [71.53, 71.92]	<0.001
Sex, No. (%)			0.27
Male	7,263 (38.0)	5,121 (37.4)	
Female	11,876 (62.0)	8,589 (62.6)	
Race			<0.001
Non-white	3,360 (17.6)	1,583 (11.5)	
White	15,779 (82.4)	12,127 (88.5)	
County Income	59464.5 [59235.7, 59693.2]	59803.3 [59528.2, 60078.4]	0.063
Current Reason for Enrollment			<0.001
Old age	15,382 (80.4)	11,644 (84.9)	
Both disability and ESRD	3,624 (18.9)	1,981 (14.5)	
ESRD	97 (0.5)	44 (0.3)	
Disability	36 (0.2)	41 (0.3)	
Dual Eligibility			<0.001
No	15,250 (79.7)	11,524 (84.1)	
Yes	3,889 (20.3)	2,186 (15.9)	
HIV/AIDS	110 (0.6)	39 (0.3)	<0.001
Septicemia, Sepsis, Systemic Inflammatory Response Syndrome/Shock	1,528 (8.0)	1,008 (7.4)	0.035
Opportunistic Infections	343 (1.8)	185 (1.4)	0.002
Metastatic Cancer and Acute Leukemia	3,261 (17.0)	1,687 (12.3)	<0.001
Lung and Other Severe Cancers	973 (5.1)	581 (4.2)	<.0001
Lymphoma and Other Cancers	1,267 (6.6)	769 (5.6)	<0.001
Colorectal, Bladder, and Other Cancers	726 (3.8)	588 (4.3)	0.024
Breast, Prostate, and Other Cancers and Tumors	2,194 (11.5)	1,530 (11.2)	0.39
Diabetes with Acute Complications	231 (1.2)	132 (1.0)	0.037
Diabetes with Chronic Complications	4,450 (23.3)	3,082 (22.5)	0.10
Diabetes without Complication	1,794 (9.4)	1,279 (9.3)	0.89
Protein-Calorie Malnutrition	1,210 (6.3)	769 (5.6)	0.008
Morbid Obesity	1,666 (8.7)	1,187 (8.7)	0.88
Other Significant Endocrine and Metabolic Disorders	2,512 (13.1)	1,688 (12.3)	0.030
End-Stage Liver Disease	350 (1.8)	244 (1.8)	0.74
Cirrhosis of Liver	267 (1.4)	159 (1.2)	0.063
Chronic Hepatitis	218 (1.1)	87 (0.6)	<0.001
Intestinal Obstruction/Perforation	986 (5.2)	699 (5.1)	0.83
Chronic Pancreatitis	135 (0.7)	86 (0.6)	0.24
Inflammatory Bowel Disease	744 (3.9)	512 (3.7)	0.48

© 2022 Li Y et al. *JAMA Network Open.*

Bone/Joint/Muscle Infections/Necrosis	527 (2.8)	474 (3.5)	<0.001
**Characteristics**	**Beneficiaries Received Part B Drug Administration at 340B hospitals only (n = 19,139)**	**Beneficiaries Received Part B Drug Administration at Non-340B hospitals only (n = 13,710)**	***P*[b]**
Rheumatoid Arthritis and Inflammatory Connective Tissue Disease	3,212 (16.8)	2,384 (17.4)	0.15
Severe Hematological Disorders	690 (3.6)	450 (3.3)	0.11
Disorders of Immunity	3,027 (15.8)	1,758 (12.8)	<0.001
Coagulation Defects and Other Specified Hematological Disorders	2,660 (13.9)	1,754 (12.8)	0.004
Dementia with Complications	261 (1.4)	206 (1.5)	0.29
Dementia without Complication	1,185 (6.2)	963 (7.0)	0.003
Drug/Alcohol Psychosis	41 (0.2)	25 (0.2)	0.52
Drug/Alcohol Dependence	926 (4.8)	607 (4.4)	0.082
Schizophrenia	284 (1.5)	157 (1.2)	0.009
Major Depressive, Bipolar, and Paranoid Disorders	2,454 (12.8)	1,606 (11.7)	0.003
Quadriplegia	149 (0.8)	96 (0.7)	0.42
Paraplegia	129 (0.7)	80 (0.6)	0.31
Spinal Cord Disorders/Injuries	348 (1.8)	218 (1.6)	0.12
Amyotrophic Lateral Sclerosis and Other Motor Neuron Disease	15 (0.1)	17 (0.1)	0.19
Cerebral Palsy	124 (0.7)	83 (0.6)	0.63
Polyneuropathy	4,757 (24.9)	3,311 (24.2)	0.14
Muscular Dystrophy	22 (0.1)	6 (0.0)	0.029
Multiple Sclerosis	435 (2.3)	234 (1.7)	<0.001
Parkinson's and Huntington's Diseases	380 (2.0)	314 (2.3)	0.058
Seizure Disorders and Convulsions	993 (5.2)	625 (4.6)	0.009
Coma, Brain Compression/Anoxic Damage	251 (1.3)	170 (1.2)	0.57
Respirator Dependence/Tracheostomy Status	175 (0.9)	98 (0.7)	0.050
Respiratory Arrest	22 (0.1)	22 (0.2)	0.27
Cardio-Respiratory Failure and Shock	1,559 (8.2)	1,143 (8.3)	0.53
Congestive Heart Failure	4,172 (21.8)	3,099 (22.6)	0.082
Acute Myocardial Infarction	645 (3.4)	521 (3.8)	0.038
Unstable Angina and Other Acute Ischemic Heart Disease	455 (2.4)	347 (2.5)	0.37
Angina Pectoris	849 (4.4)	595 (4.3)	0.68
Specified Heart Arrhythmias	3,991 (20.9)	3,124 (22.8)	<0.001
Cerebral Hemorrhage	235 (1.2)	189 (1.4)	0.23
Ischemic or Unspecified Stroke	1,058 (5.5)	770 (5.6)	0.73
Hemiplegia/Hemiparesis	650 (3.4)	447 (3.3)	0.50
Monoplegia, Other Paralytic Syndromes	50 (0.3)	32 (0.2)	0.62
Atherosclerosis of the Extremities with Ulceration or Gangrene	214 (1.1)	211 (1.5)	<0.001
Vascular Disease with Complications	993 (5.2)	688 (5.0)	0.49

© 2022 Li Y et al. *JAMA Network Open.*

Vascular Disease	4,683 (24.5)	3,334 (24.3)	0.75

Abbreviations: ESRD, end-stage renal disease
[a]The exhibit shows the results of the beneficiary characteristics by hospital 340B status among beneficiaries who have only gone to one type of hospital (n =32,849).
[b]P values were generated from chi-square tests or t-tests.

© 2022 Li Y et al. *JAMA Network Open.*

eTable 2. Association of Each Beneficiary Characteristic and Drug Spending After Controlling for Other Beneficiary Characteristics, 2017 (n = 35,364)[a]

Characteristics	Estimate (95% CI)	T Value	*P* value
Age	-174 [-213, -135]	-8.68	<.001
Sex<br>Male	Reference group		
Female	-725 [-1324, -125]	-2.37	0.02
Race			
White	Reference group		
Non-white	-111 [-932, 710]	-0.27	0.79
County Income (Per $1,000)	27 [17, 44]	3.15	0.002
Current Reason for Enrollment			
Both disability and ESRD	Reference group		
Old age	6526 [433, 12619]	2.1	0.04
Disability	7912 [1863, 13961]	2.56	0.01
ESRD	12611 [5429, 19792]	3.44	<.001
Dual Eligibility			
No	Reference group		
Yes	-1,241 [-1,960, -533]	-3.39	<.001
HIV/AIDS	4797 [646, 8948]	2.26	0.02
Septicemia, Sepsis, Systemic Inflammatory Response Syndrome/Shock	-578 [-1772, 615]	-0.95	0.34
Opportunistic Infections	-3144 [-5373, -914]	-2.76	0.006
Metastatic Cancer and Acute Leukemia	15420 [14574, 16266]	35.73	<.001
Lung and Other Severe Cancers	8861 [7513, 10209]	12.88	<.001
Lymphoma and Other Cancers	13749 [12554, 14944]	22.55	<.001
Colorectal, Bladder, and Other Cancers	-136 [-1582, 1311]	-0.18	0.85
Breast, Prostate, and Other Cancers and Tumors	-93 [-1007, 821]	-0.2	0.84
Diabetes with Acute Complications	-1096 [-3820, 1629]	-0.79	0.43
Diabetes with Chronic Complications	-1236 [-2036, -437]	-3.03	0.002
Diabetes without Complication	311 [-658, 1279]	0.63	0.53
Protein-Calorie Malnutrition	-2015 [-3287, -742]	-3.1	0.002
Morbid Obesity	1690 [648, 2732]	3.18	0.002
Other Significant Endocrine and Metabolic Disorders	2828 [1894, 3761]	5.94	<.001
End-Stage Liver Disease	-5607 [-7720, -3494]	-5.2	<.001
Cirrhosis of Liver	-2871 [-5325, -418]	-2.29	0.02
Chronic Hepatitis	-2253 [-5123, 616]	-1.54	0.12
Intestinal Obstruction/Perforation	-1230 [-2539, 79]	-1.84	0.07
Chronic Pancreatitis	-576 [-3993, 2841]	-0.33	0.74
Inflammatory Bowel Disease	6567 [5101, 8033]	8.78	<.001
Bone/Joint/Muscle Infections/Necrosis	-692 [-2450, 1066]	-0.77	0.44
Rheumatoid Arthritis and Inflammatory Connective Tissue Disease	4517 [3755, 5279]	11.62	<.001
Severe Hematological Disorders	9856 [8323, 11389]	12.6	<.001

© 2022 Li Y et al. *JAMA Network Open.*

Characteristics	Estimate (95% CI)	T Value	*P*
Disorders of Immunity	5057 [4191, 5924]	11.44	<.001
Coagulation Defects and Other Specified Hematological Disorders	2582 [1717, 3447]	5.85	<.001
Dementia with Complications	583 [-1822, 2987]	0.47	0.63
Dementia without Complication	-1070 [-2252, 113]	-1.77	0.08
Drug/Alcohol Psychosis	-2676 [-8863, 3511]	-0.85	0.40
Drug/Alcohol Dependence	-2961 [-4323, -1599]	-4.26	<.001
Schizophrenia	-2020 [-4523, 483]	-1.58	0.11
Major Depressive, Bipolar, and Paranoid Disorders	-1736 [-2624, -848]	-3.83	<.001
Quadriplegia	-1528 [-4955, 1898]	-0.87	0.38
Paraplegia	-2413 [-6009, 1183]	-1.32	0.19
Spinal Cord Disorders/Injuries	-2203 [-4335, -72]	-2.03	0.04
Amyotrophic Lateral Sclerosis and Other Motor Neuron Disease	9611 [791, 18431]	2.14	0.03
Cerebral Palsy	-5330 [-8959, -1701]	-2.88	0.004
Polyneuropathy	3492 [2775, 4209]	9.55	<.001
Muscular Dystrophy	26717 [17181, 36253]	5.49	<.001
Multiple Sclerosis	12041 [10003, 14079]	11.58	<.001
Parkinson's and Huntington's Diseases	-331 [-2286, 1623]	-0.33	0.74
Seizure Disorders and Convulsions	-1303 [-2660, 55]	-1.88	0.06
Coma, Brain Compression/Anoxic Damage	-192 [-2716, 2331]	-0.15	0.88
Respirator Dependence/Tracheostomy Status	-1881 [-5032, 1270]	-1.17	0.24
Respiratory Arrest	726 [-6918, 8369]	0.19	0.85
Cardio-Respiratory Failure and Shock	319 [-846, 1483]	0.54	0.59
Congestive Heart Failure	694 [-104, 1492]	1.7	0.09
Acute Myocardial Infarction	-1876 [-3447, -305]	-2.34	0.02
Unstable Angina and Other Acute Ischemic Heart Disease	-2564 [-4395, -732]	-2.74	0.006
Angina Pectoris	-2182 [-3555, -809]	-3.12	0.002
Specified Heart Arrhythmias	-471 [-1218, 277]	-1.23	0.22
Cerebral Hemorrhage	983 [-1766, 3733]	0.7	0.48
Ischemic or Unspecified Stroke	37 [-1321, 1396]	0.05	0.96
Hemiplegia/Hemiparesis	-1630 [-3391, 131]	-1.81	0.07
Monoplegia, Other Paralytic Syndromes	-673 [-6126, 4781]	-0.24	0.81
Atherosclerosis of the Extremities with Ulceration or Gangrene	-2149 [-4789, 492]	-1.59	0.11
Vascular Disease with Complications	-715 [-2029, 599]	-1.07	0.29
Vascular Disease	87 [-615, 788]	0.24	0.81

Abbreviations: CI, confidence interval; ESRD, end-stage renal disease
[a]The exhibit shows the results of the beneficiary-level multiple linear regression analyses. Reference group for each clinical condition is beneficiaries without the clinical condition.

© 2022 Li Y et al. *JAMA Network Open.*
